# Regional Management Units for Marine Turtles: A Novel Framework for Prioritizing Conservation and Research across Multiple Scales

**DOI:** 10.1371/journal.pone.0015465

**Published:** 2010-12-17

**Authors:** Bryan P. Wallace, Andrew D. DiMatteo, Brendan J. Hurley, Elena M. Finkbeiner, Alan B. Bolten, Milani Y. Chaloupka, Brian J. Hutchinson, F. Alberto Abreu-Grobois, Diego Amorocho, Karen A. Bjorndal, Jerome Bourjea, Brian W. Bowen, Raquel Briseño Dueñas, Paolo Casale, B. C. Choudhury, Alice Costa, Peter H. Dutton, Alejandro Fallabrino, Alexandre Girard, Marc Girondot, Matthew H. Godfrey, Mark Hamann, Milagros López-Mendilaharsu, Maria Angela Marcovaldi, Jeanne A. Mortimer, John A. Musick, Ronel Nel, Nicolas J. Pilcher, Jeffrey A. Seminoff, Sebastian Troëng, Blair Witherington, Roderic B. Mast

**Affiliations:** 1 International Union for Conservation of Nature (IUCN)/SSC Marine Turtle Specialist Group – Burning Issues Working Group, Arlington, Virginia, United States of America; 2 Global Marine Division, Conservation International, Arlington, Virginia, United States of America; 3 Center for Marine Conservation, Duke University, Beaufort, North Carolina, United States of America; 4 Marine Geospatial Ecology Laboratory, Duke University, Durham, North Carolina, United States of America; 5 Department of Biology, Archie Carr Center for Sea Turtle Research, University of Florida, Gainesville, Florida, United States of America; 6 Ecological Modelling Services, Pty Ltd, University of Queensland, Brisbane, Australia; 7 Unidad Académica Mazatlán, Instituto de Ciencias del Mar y Limnología, Universidad Nacional Autónoma de México, Mazatlán, Sinaloa, México; 8 Centro de Investigación para el Medio Ambiente y Desarrollo, Cali, Colombia; 9 Laboratoire Ressources Halieutiques, IFREMER, Ile Reunion, France; 10 Hawaii Institute of Marine Biology, Kaneohe, Hawaii, United States of America; 11 Banco de Información sobre Tortugas Marinas (BITMAR), Unidad Mazatlán, Instituto de Ciencias del Mar y Limnología, Universidad Nacional Autónoma de México, Mazatlán, Sinaloa, México; 12 Department of Biology and Biotechnology “Charles Darwin,” University of Rome “La Sapienza,” Rome, Italy; 13 World Wildlife Fund (WWF) Mediterranean Turtle Programme, World Wildlife Fund-Italy, Rome, Italy; 14 Department of Endangered Species Management, Wildlife Institute of India, Dehradun, Uttarakhand, India; 15 World Wildlife Fund-Mozambique, Maputo, Mozambique; 16 Southwest Fisheries Science Center, National Marine Fisheries Service, National Oceanic and Atmospheric Administration (NOAA), La Jolla, California, United States of America; 17 Karumbé, Montevideo, Uruguay; 18 Association RENATURA, Albens, France, and Pointe-Noire, Congo; 19 Laboratoire d'Ecologie, Systématique et Evolution, Université Paris-Sud, Orsay, France; 20 North Carolina Wildlife Resources Commission, Beaufort, North Carolina, United States of America; 21 School of Earth and Environmental Sciences, James Cook University, Townsville, Australia; 22 Projeto Tamar-ICMBio/Fundação Pro Tamar, Salvador, Bahía, Brazil; 23 Department of Ecology, Institute of Biology, Universidade do Estado do Rio de Janeiro, Rio de Janeiro, Brazil; 24 Department of Biology, University of Florida, Gainesville, Florida, United States of America; 25 Virginia Institute of Marine Sciences, College of William and Mary, Gloucester Point, Virginia, United States of America; 26 School of Environmental Sciences, Nelson Mandela Metropolitan University, Summerstrand Campus, South Africa; 27 Marine Research Foundation, Sabah, Malaysia; 28 Marine Turtle Ecology and Assessment Program, Southwest Fisheries Science Center, NOAA-National Marine Fisheries Service, La Jolla, California, United States of America; 29 Department of Animal Ecology, Lund University, Lund, Sweden; 30 Scientific Advisory Committee, Sea Turtle Conservancy, Gainesville, Florida, United States of America; 31 Florida Fish and Wildlife Conservation Commission, Melbourne Beach, Florida, United States of America; NOAA/NMFS/SWFSC, United States of America

## Abstract

**Background:**

Resolving threats to widely distributed marine megafauna requires definition of the geographic distributions of both the threats as well as the population unit(s) of interest. In turn, because individual threats can operate on varying spatial scales, their impacts can affect different segments of a population of the same species. Therefore, integration of multiple tools and techniques — including site-based monitoring, genetic analyses, mark-recapture studies and telemetry — can facilitate robust definitions of population segments at multiple biological and spatial scales to address different management and research challenges.

**Methodology/Principal Findings:**

To address these issues for marine turtles, we collated all available studies on marine turtle biogeography, including nesting sites, population abundances and trends, population genetics, and satellite telemetry. We georeferenced this information to generate separate layers for nesting sites, genetic stocks, and core distributions of population segments of all marine turtle species. We then spatially integrated this information from fine- to coarse-spatial scales to develop nested envelope models, or Regional Management Units (RMUs), for marine turtles globally.

**Conclusions/Significance:**

The RMU framework is a solution to the challenge of how to organize marine turtles into units of protection above the level of nesting populations, but below the level of species, within regional entities that might be on independent evolutionary trajectories. Among many potential applications, RMUs provide a framework for identifying data gaps, assessing high diversity areas for multiple species and genetic stocks, and evaluating conservation status of marine turtles. Furthermore, RMUs allow for identification of geographic barriers to gene flow, and can provide valuable guidance to marine spatial planning initiatives that integrate spatial distributions of protected species and human activities. In addition, the RMU framework — including maps and supporting metadata — will be an iterative, user-driven tool made publicly available in an online application for comments, improvements, download and analysis.

## Introduction

Geospatial characterization of commercially important or conservation-dependent marine species provides crucial input for resource management in multi-use situations, as is currently described by ecosystem-based marine spatial planning [1]. In particular, linking impacts of various threats to widely distributed marine megafauna populations (e.g. mammals, birds, turtles, sharks) requires description of overlaps between threats and the population segment of interest. Furthermore, widespread marine species often exhibit inter-population variation in life history traits and population dynamics that warrant population-specific management schemes [2], [3].

However, resolution of population units for conservation is not always straightforward, and requires clear understanding of conservation objectives, as well as the natural history of – and threats to – the species of interest [4]. For example, evolutionarily significant units (ESUs) were first described to include sufficient genetic diversity to retain evolutionary potential, and thus address long-term conservation issues as well as historical population trends [5], [6]. In contrast, population segments or management units (MUs) are functionally independent (i.e. exhibit distinct demographic processes), can be characterized using various tools or indicators, such as genetic markers, life history traits, behavior, or morphology, and are appropriate short-term targets for conservation [5]. A major challenge to prioritization schemes arises when multiple population segments meet the criteria of a MU, each deserving specially designed conservation strategies.

Six of the seven marine turtle species are categorized as Vulnerable, Endangered, or Critically Endangered globally by the *IUCN Red List of Threatened Species* [7], but threats on regional scales can differentially affect life-stages of the same populations. Similar to other long-lived marine vertebrates, marine turtles occupy broad geographic ranges including separate breeding and feeding areas utilized by adults, and in some cases geographically distinct ontogenetic habitats for immature life stages, with different levels of population overlap at each stage [8]. Furthermore, marine turtles have complex population structures characterized by female nesting site fidelity, male-mediated gene flow, and population overlap during migrations and in developmental habitats, with the degree of genetic population structuring increasing with life stages (see [9] for review). Understanding the complex relationships among various nesting sites, nurseries, and foraging areas, particularly in the context of variation in environmental conditions, is crucial to quantifying population-level impacts of anthropogenic threats, as well as to designing effective conservation responses to these threats [10]–[12]. However, these complexities in population structures, habitat use, environmental factors, and life-stage-specific threats have confounded the definition of marine turtle MUs.

Molecular genetic analyses are often used to describe population structures and, by extension, to define MUs [4], [5], [13]. Due to complex population structure and life history of marine turtles, different molecular analyses can be applied to determine genetic stock structure for different demographic segments of a population. Maternally inherited mitochondrial DNA (mtDNA) is useful for resolving nest site fidelity and homing behavior [14], [15]. In addition to defining nesting populations (i.e. one level of MUs), this genetic marker is also useful for resolving maternal origin of both males and females at various life stages and feeding habitats [9].

Nesting females typically demonstrate philopatry to nesting areas, and both males and females can be philopatric to breeding areas adjacent to a nesting beach [16]. However, males do not restrict mating efforts to their ancestral breeding area, and apparently copulate with females in coastal feeding habitat or migratory corridors as well, where they encounter females from other regional nesting populations. The result, registered in biparentally-inherited nuclear DNA (nDNA), is that regional nesting colonies are connected by this male-mediated gene flow [2], [17]–[18]. Because nDNA reflects contributions of both males and females, analyses of nDNA markers (e.g. microsatellites) can resolve breeding or reproductive stocks that encompass multiple mtDNA-defined nesting stocks [9], [19].

Genetic analyses clearly contribute to the resolution of MUs, but are not always sufficient for this purpose; such analyses can indicate that genetic population structure exists, but usually cannot resolve the boundaries of that structure [4], [20]. Unique identifier tags applied to marine turtles at various life stages and in various habitats via mark-recapture monitoring programs can illuminate migration routes and connectivity of individual animals between habitats [21]–[23]. Furthermore, the advent of electronic tracking technology, specifically satellite telemetry and remote sensing, has facilitated an exponential increase in understanding of marine turtle movements, behaviors, biology, and conservation concerns (see [24] for review). Therefore, integration of multiple tools and techniques, including site-based monitoring (e.g. nesting beaches, foraging areas), genetic analyses, mark-recapture studies, and telemetry, especially when supplemented with information on threats and influence of environmental conditions [25], can facilitate robust definitions of MUs for marine turtles at multiple scales to address different management and research challenges.

In response to these issues, we compiled, collated, and georeferenced available information on marine turtle biogeography – including individual nesting sites, genetic stocks, and geographic distributions based on monitoring research – to develop multi-scale Regional Management Units (RMUs). These RMUs spatially integrate sufficient information to account for complexities in marine turtle population structures, and thus provide a flexible, dynamic framework for evaluating threats, identifying high diversity areas, highlighting data gaps, and assessing conservation status of marine turtles.

## Methods

The IUCN's Marine Turtle Specialist Group convened the Burning Issues Working Group (MTSG-BI) for two meetings (August 2008 and September 2009) of marine turtle experts from around the world who represented government agencies, non-governmental organizations, and academic institutions. The primary objective of these meetings was to develop a process for evaluating and prioritizing the conservation status of marine turtle populations worldwide. In this context, the MTSG-BI developed Regional Management Units (RMUs) as the framework for this evaluation and prioritization process.

To generate RMUs for marine turtles, we collated and georeferenced available data from more than 1,200 papers, reports, abstracts, and other sources (available for download at http://tinyurl.com/29w4kbf), extracting information on nesting sites, population genetics, tag returns, and satellite telemetry, as well as other relevant natural history and biogeography. We then spatially integrated this information in ArcMap 9.3 (ESRI, Redlands, CA USA) from fine (i.e. points with geographic coordinates for nesting sites) to coarse (i.e. polygon shapefiles for distributions) spatial scales. We constructed separate layers according to distinct biological/spatial scales, including a layer for nesting sites (i.e. individual nesting beaches), and two layers for genetic stocks (i.e. a layer each for maternally inherited mtDNA and biparentally inherited nDNA, respectively). Finally, all finer-scale levels were nested within the known core geographic distributions of these population units, according to satellite telemetry and tag return data. In this way, all nesting sites that were sampled and analyzed for genetic studies were represented within genetic stock layers, and defined genetic stocks were represented within RMU layers, such that information shared across scales was retained in each relevant layer. We also compiled available information on population sizes (i.e. annual numbers of nesting females) and trends (i.e. change in annual numbers of nesting females over time) at each spatial/biological scale. Although considerable uncertainty exists in estimates of population sizes and trends for marine turtles among sites globally, we did not attempt to standardize or improve estimates; we urge caution in interpretation of these metadata. This “nested envelope” approach allowed for metadata to be arranged within and across biologically defined spatial scales.

### Nesting Sites

**Figure 1:**
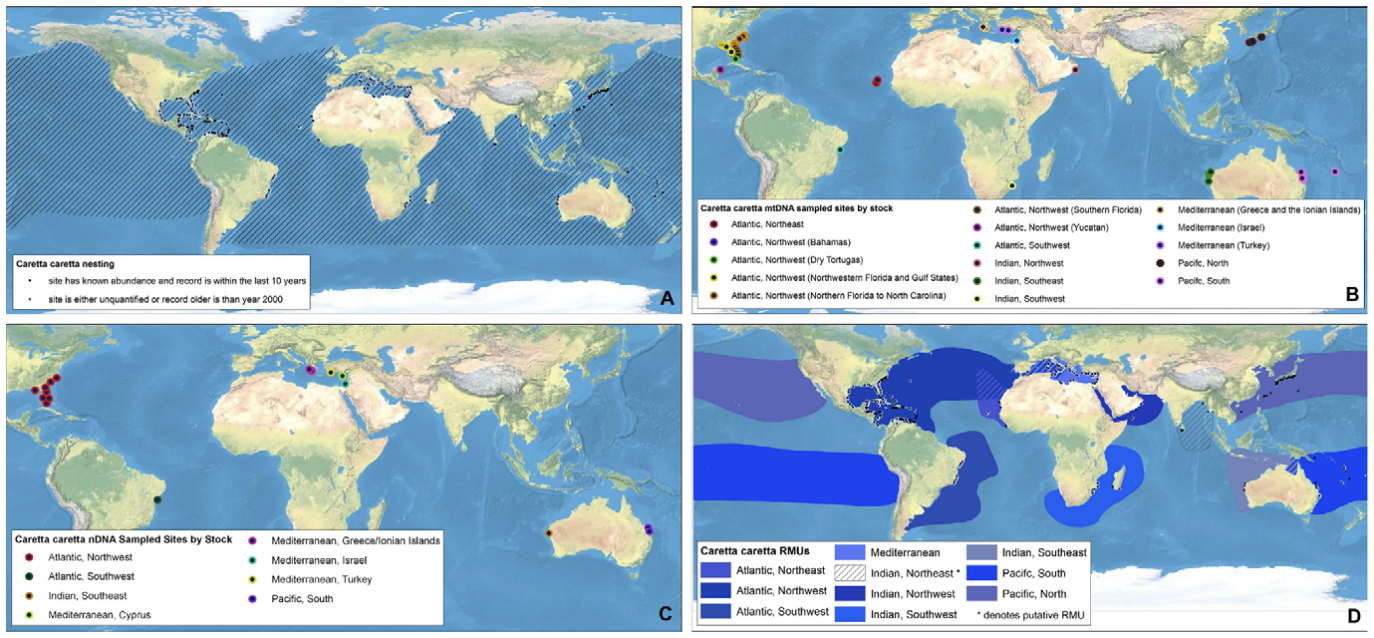
Multi-scale Regional Management Units for loggerhead turtles *Caretta caretta*. Individual maps are presented for A) global nesting sites and in-water distributions (global distributions for each species represented by hatching); B) mitochondrial (mtDNA) genetic stocks; C) nuclear genetic (nDNA) stocks; and D) Regional Management Units (shown with nesting sites). Nesting sites that were unquantified or have no count values reported since 2000 are represented by black squares, whereas nesting sites for which count data are available are represented by a colored circle. For genetic stock maps (Figs. 1B and C), each symbol represents a different site that was sampled for genetic analyses, while each color represents a distinct genetic stock. Putative RMUs (see text for description) are noted by asterisks in the figure legends, and are colorless. Data shown are contained in metadata tables (Appendix S2).

**Figure 2:**
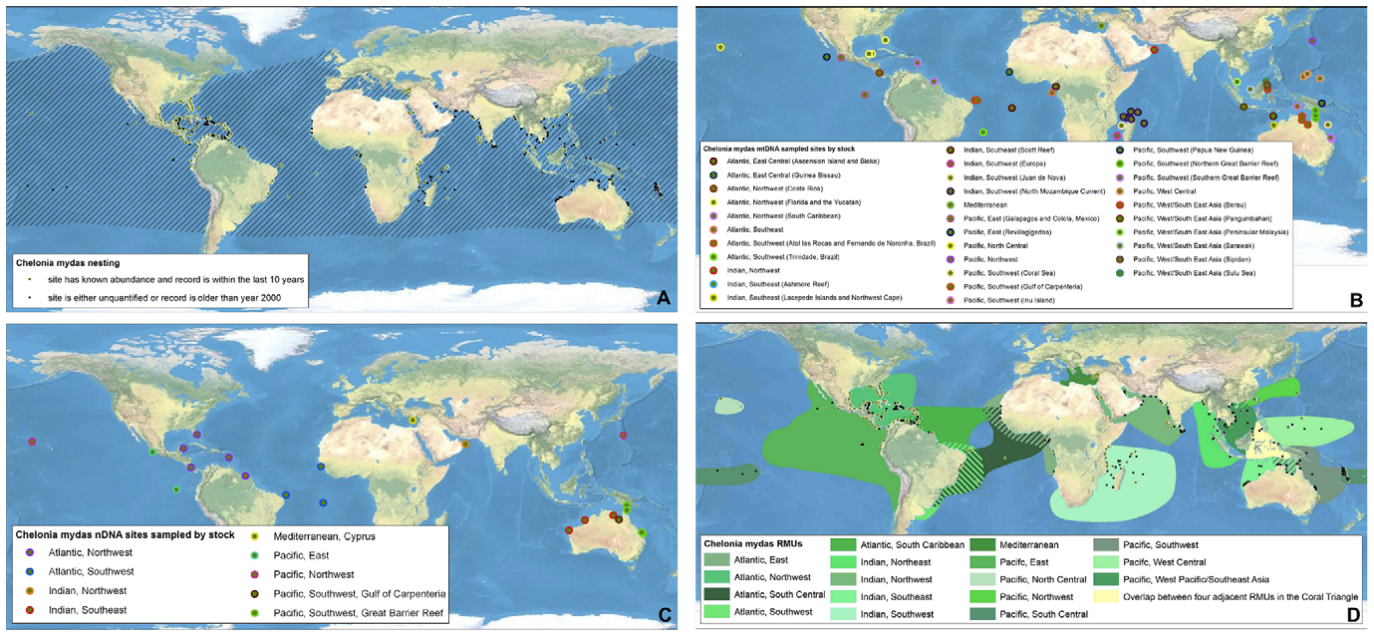
Multi-scale Regional Management Units for green turtles *Chelonia mydas*. Individual maps are presented for A) global nesting sites and in-water distributions (global distributions for each species represented by hatching); B) mitochondrial (mtDNA) genetic stocks; C) nuclear genetic (nDNA) stocks; and D) Regional Management Units (shown with nesting sites). Nesting sites that were unquantified or have no count values reported since 2000 are represented by black squares, whereas nesting sites for which count data are available are represented by a colored circle. For genetic stock maps (Figs. 2B and C), each symbol represents a different site that was sampled for genetic analyses, while each color represents a distinct genetic stock. Data shown are contained in metadata tables (Appendix S2).

**Figure 3:**
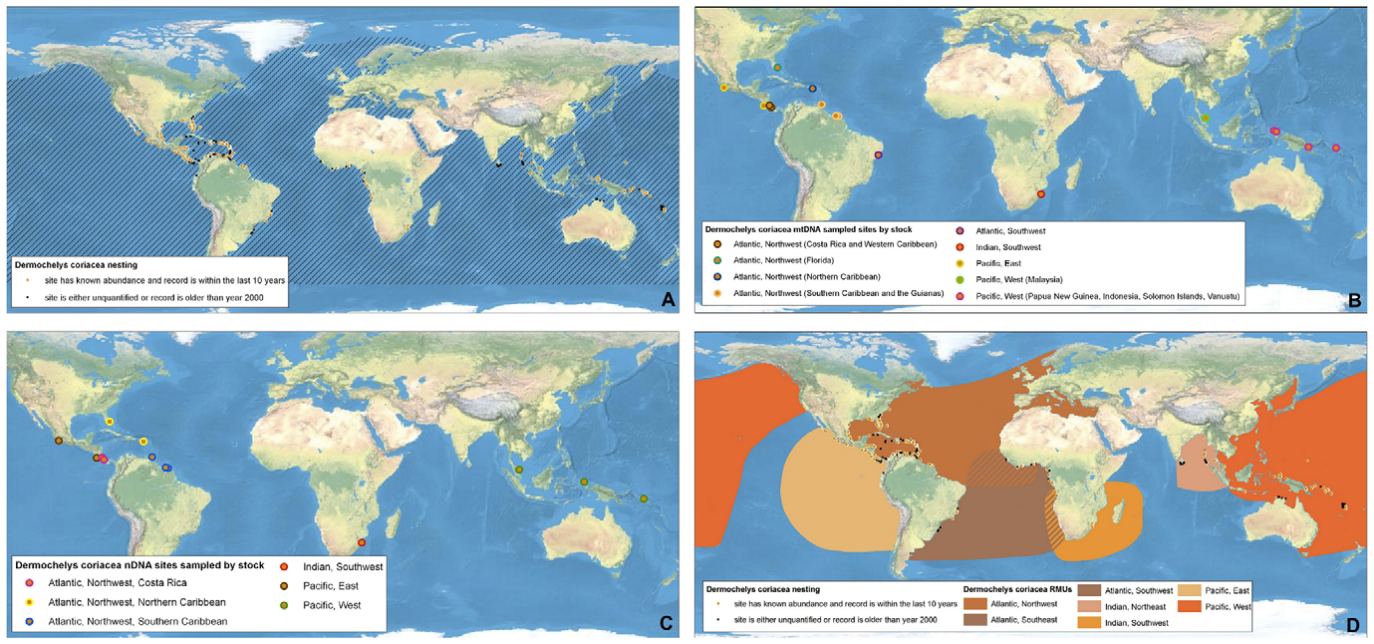
Multi-scale Regional Management Units for leatherback turtles *Dermochelys coriacea*. Individual maps are presented for A) global nesting sites and in-water distributions (global distributions for each species represented by hatching); B) mitochondrial (mtDNA) genetic stocks; C) nuclear genetic (nDNA) stocks; and D) Regional Management Units (shown with nesting sites). Nesting sites that were unquantified or have no count values reported since 2000 are represented by black squares, whereas nesting sites for which count data are available are represented by a colored circle. For genetic stock maps (Figs. 3B and C), each symbol represents a different site that was sampled for genetic analyses, while each color represents a distinct genetic stock. Data shown are contained in metadata tables (Appendix S2).

**Figure 4:**
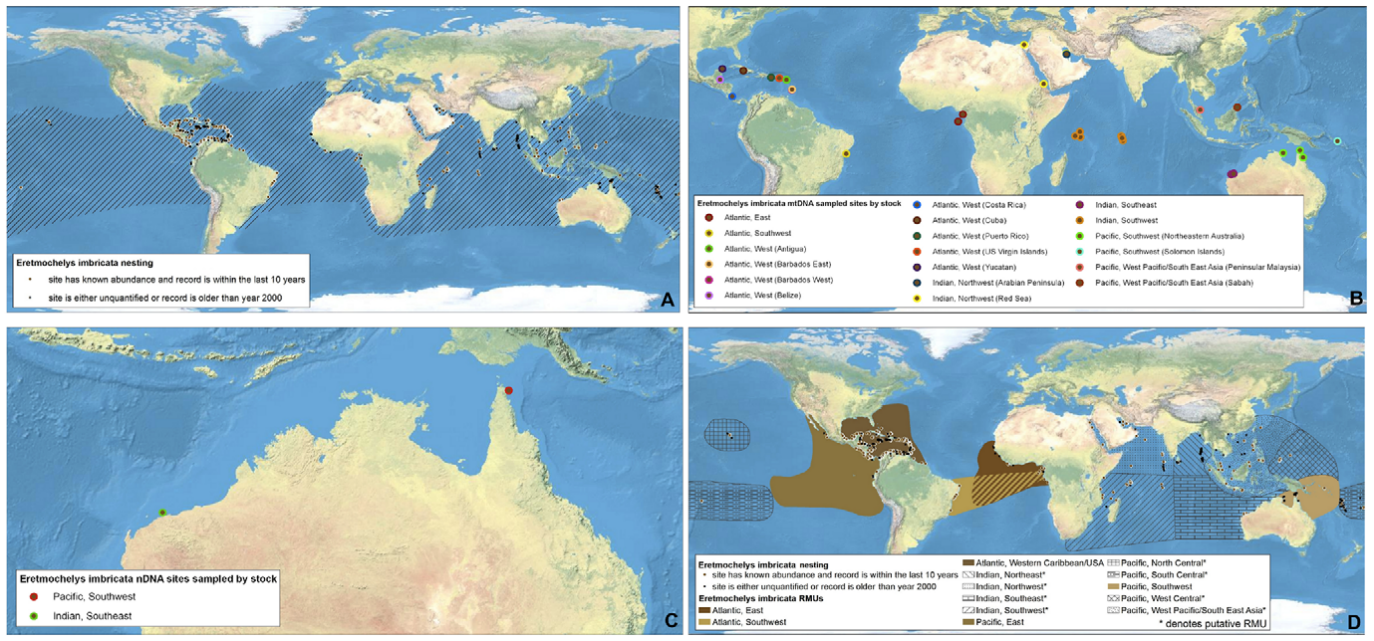
Multi-scale Regional Management Units for hawksbill turtles *Eretmochelys imbricata*. Individual maps are presented for A) global nesting sites and in-water distributions (global distributions for each species represented by hatching); B) mitochondrial (mtDNA) genetic stocks; C) nuclear genetic (nDNA) stocks; and D) Regional Management Units (shown with nesting sites). Nesting sites that were unquantified or have no count values reported since 2000 are represented by black squares, whereas nesting sites for which count data are available are represented by a colored circle. For genetic stock maps (Figs. 4B and C), each symbol represents a different site that was sampled for genetic analyses, while each color represents a distinct genetic stock. Putative RMUs (see text for description) are noted by asterisks in the figure legends, and are colorless. Data shown are contained in metadata tables (Appendix S2).

**Figure 5:**
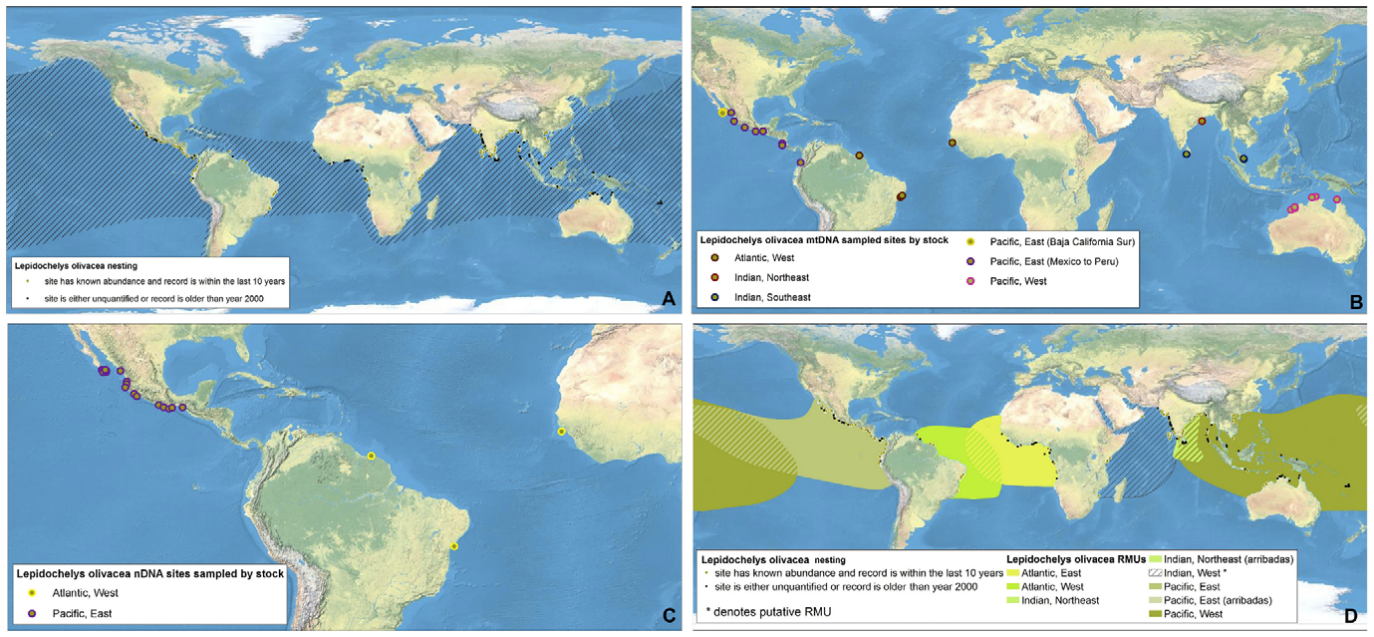
Multi-scale Regional Management Units for olive ridley turtles *Lepidochelys olivacea*. Individual maps are presented for A) global nesting sites and in-water distributions (global distributions for each species represented by hatching); B) mitochondrial (mtDNA) genetic stocks; C) nuclear genetic (nDNA) stocks; and D) Regional Management Units (shown with nesting sites). Nesting sites that were unquantified or have no count values reported since 2000 are represented by black squares, whereas nesting sites for which count data are available are represented by a colored circle. For genetic stock maps (Figs. 5B and C), each symbol represents a different site that was sampled for genetic analyses, while each color represents a distinct genetic stock. Putative RMUs (see text for description) are noted by asterisks in the figure legends, and are colorless. Data shown are contained in metadata tables (Appendix S2).

**Figure 6:**
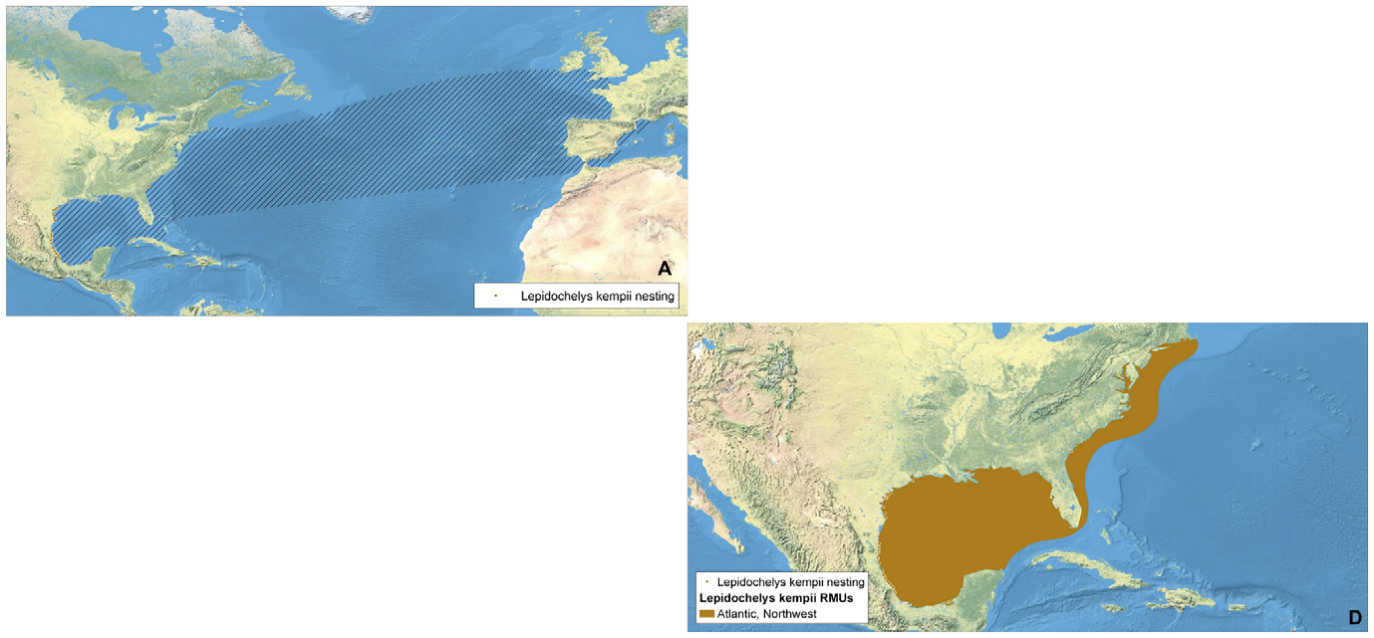
Multi-scale Regional Management Units for Kemp's ridley turtles *Lepidochelys kempii*. Individual maps are presented for A) global nesting sites and in-water distributions (global distributions for each species represented by hatching); and D) Regional Management Units (shown with nesting sites). Nesting sites that were unquantified or have no count values reported since 2000 are represented by black squares, whereas nesting sites for which count data are available are represented by a colored circle. Putative RMUs (see text for description) are noted by asterisks in the figure legends, and are colorless. Note: There are no genetics maps (Panels B or C) for *L. kempii* because all individuals are presumed to belong to same stock and RMU. Data shown are contained in metadata tables (Appendix S2).

**Figure 7:**
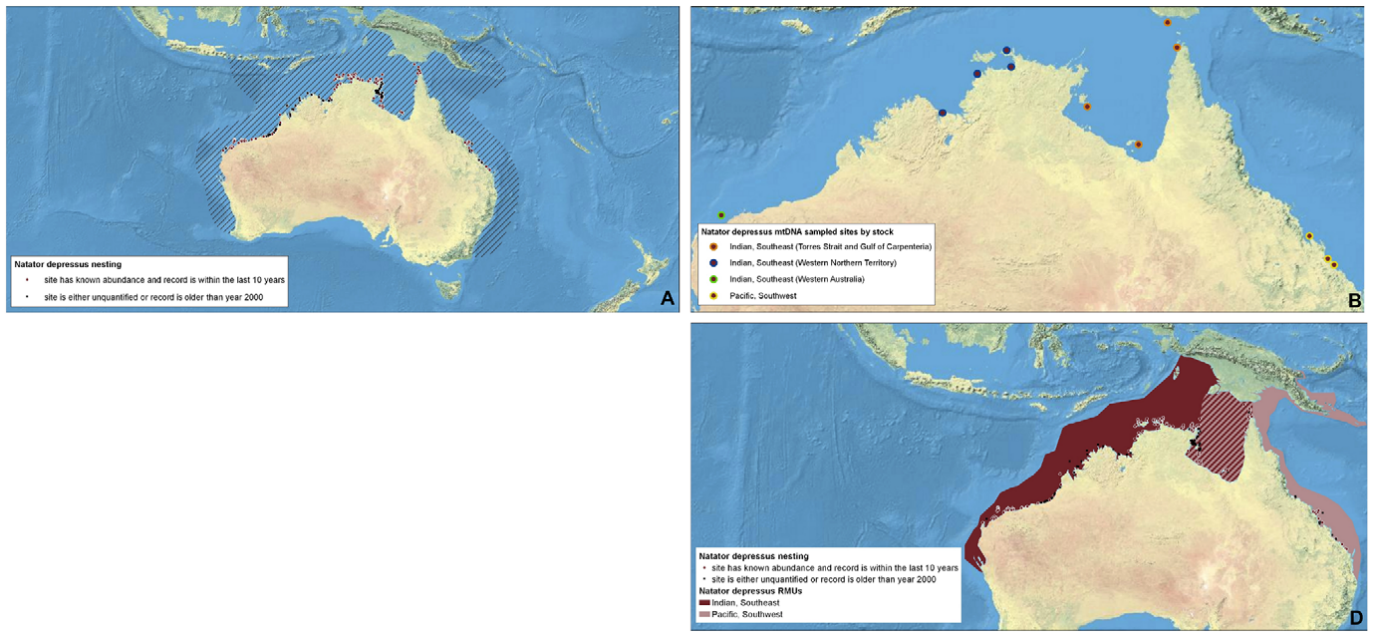
Multi-scale Regional Management Units for flatback turtles *Natator depressus*. Individual maps are presented for A) global nesting sites and in-water distributions (global distributions for each species represented by hatching); B) mitochondrial (mtDNA) genetic stocks; and D) Regional Management Units (shown with nesting sites). Nesting sites that were unquantified or have no count values reported since 2000 are represented by black squares, whereas nesting sites for which count data are available are represented by a colored circle. For genetic stock maps (Fig. 7B), each symbol represents a different site that was sampled for genetic analyses, while each color represents a distinct genetic stock. Note: There is no map for *N. depressus* nDNA stocks (panel C, due to lack of available data). Data shown are contained in metadata tables (Appendix S2).

We defined a nesting site as a beach or beaches with confirmed marine turtle nesting activity monitored or analyzed as a singular site by the groups or individuals providing or publishing the nesting data. To compile and georeference nesting sites globally for all species, we used the State of the World's Sea Turtles – SWOT (www.seaturtlestatus.org) database, which relies on a global network of researchers who voluntarily contribute annual nesting data. We augmented the SWOT database with published information. We filtered all nesting sites to distinguish sites with confirmed, quantified nesting activity (i.e. counts) from those without quantified counts since 2000. Across species globally, we compiled more than 4,200 nesting sites, ranging from 30 for Kemp's ridleys (*Lepidochelys kempii*) to 1,337 for hawksbill turtles (*Eretmochelys imbricata*) (Table 1; Figs. 1, 2, 3, 4, 5, 6, 7, panel A). In accordance with data sharing protocols, metadata for SWOT nesting sites are not provided here. However, all SWOT nesting data can be viewed at http://seamap.env.duke.edu/swot, and a complete list of SWOT data providers is provided in Appendix S1.

**Table 1:** Summary of number of nesting sites, genetic stocks, and RMUs identified for all marine turtle species.

species	no. nesting sites	no. mtDNA stocks	no. nDNA stocks	no. RMUs(no. putative RMUs)
*Caretta caretta*	626	15	8	10 (1)
*Chelonia mydas*	935	33	9	17
*Demochelys coriacea*	652	9	6	7
*Eretmochelys imbricata*	1,337	19	2	13 (7)
*Lepidochelys kempii*	30	1*	1*	1
*Lepidochelys olivacea*	426	6	2	8 (1)
*Natator depressus*	287	4	ND	2
TOTAL	4,293	87	28	58 (9)

ND: no data. Putative RMUs were described for regions with available nesting data but no associated data on genetics or distributions. (

*denotes that although nesting sites have not been sampled for genetics, all *L. kempii* individuals are presumed to belong to same stock and a single RMU.)

### Genetic Stocks

We compiled and georeferenced genetic stock information from available mtDNA and nDNA studies by constructing shapefiles that included all individual nesting sites upon which the genetic sampling and analyses were based. We identified 87 distinct mtDNA and 28 nDNA reported genetic stocks globally across marine turtle species (Table 1; Figs. 1, 2, 3, 4, 5, 6, 7, panels B and C; Appendix S2). While recognizing the diversity of analytical techniques – particularly the number and types of genetic markers, sample sizes, and statistical approaches – used to determine genetic stocks of marine turtles, we accepted genetic stock definitions as described by the original sources, rather than attempting to standardize or prioritize methodologies or interpretations. However, in cases where more recent studies clarified or contradicted earlier studies, we based our dataset on the results of the updated studies.

### Regional Management Units

We generated polygons representing RMUs for all species of marine turtles, or geographically explicit population segments based on geographic boundaries to distributions derived from studies on genetics, tag returns, satellite telemetry, and other data (Figs. 1, 2, 3, 4, 5, 6, 7, panel D; Table S1; Appendix S2). RMUs were meant to encompass multiple nesting sites, mtDNA-defined nesting populations, and nDNA-defined breeding populations to reflect shared geographic distribution among conspecific marine turtles in the same region. Thus, RMUs do not represent complete geographic distributions of species on global or regional scales, but rather distributions that are anchored to landmasses by known nesting site(s) and/or genetic stock origins and defined by biogeographical information.

Specifically, we defined the boundaries of RMU polygons by generating termini directly from published satellite tracks, tag returns, or other data sources. These polygons were then digitized or imported into ArcGIS 9.3 either using the georeference tool in the ArcMap Toolbox, or based on text descriptions. In the absence of sufficient information, boundaries were further refined by MTSG experts. To allow clear distinctions between RMUs and complete global distributions, we also generated global distribution polygons for all species (Figs. 1, 2, 3, 4, 5, 6, 7, panel A; Appendix S2). These global distributions are coarse geographical representations of documented occurrence patterns – bounded by maximum extents – for each species. This process was generally similar to that used to generate species range maps for other taxonomic groups, such as those produced by the IUCN *Red List of Threatened Species* (http://www.iucnredlist.org/technical-documents/spatial-data). We acknowledge that this distinction means that RMU designations might not address threats occurring outside RMU boundaries but within the distribution of a species, but RMUs nonetheless encompass known core habitats and life stages. However, the important point is that whereas global distributions simply display broad geographic ranges for each species (including RMUs), the RMUs themselves provide refined spatial guidance for conservation strategies.

Olive ridley turtles (*Lepidochelys olivacea*) (along with their congener, Kemp's ridleys), are unique among marine turtles for their polymorphic nesting behavior; i.e. synchronous mass nesting at particular beaches (termed *arribadas*) and disperse, asynchronous or solitary nesting at the rest of the species' nesting beaches. Therefore, we established separate RMUs for *arribadas* and solitary nesters in regions where both behaviors occur (i.e. East Pacific Ocean, East Indian Ocean). Although the geographic boundaries of these RMUs are identical within a region, this dichotomy accurately reflects differences in population abundances and trends between the two behaviors [26].

In regions where nesting sites were known for certain species, but no other biological information (e.g. genetics or distributions) was available, we developed ‘putative RMUs’ so that no region-species combination was excluded. As with all RMUs, these putative RMUs will require modification as new information becomes available, but in the meantime, they represent obvious research and reporting priorities.

## Results and Discussion

We identified 58 RMUs among the seven marine turtle species worldwide, ranging from a single RMU for Kemp's ridleys to 17 RMUs for green turtles (*Chelonia mydas*) (Figs. 1, 2, 3, 4, 5, 6, 7; Table S1, Appendix S2). The RMU framework is essentially a set of nested envelope models intended for multiple research and management applications. However, the efficacy of applications using RMUs depends on the accuracy and quality of the data contained in the files; data exist that we were unable to acquire and incorporate, and current designations of genetic stocks and geographic distributions are subject to change with new and improved analyses. For example, genetic analyses using new markers, larger sample sizes, and broader geographic sampling could reveal new or more nuanced stock structures, especially because insufficient sampling is always a major detractor to explanatory power of these analyses [20], [27], [28]. Furthermore, because RMU boundaries are sometimes based on reports that contain relatively few localizations, some of which might be species misidentifications [29], improved data quality will help to resolve the geographic extents of RMUs.

Along these lines, because the metadata we compiled is derived from publicly available sources, the RMUs themselves will change with new, refined data. To facilitate iterative improvement of RMUs, we have made all map files (Figs. 1, 2, 3, 4, 5, 6, 7) and metadata associated with each layer (Appendix S2) available for comments and suggested edits, as well as download and analysis (in accordance with relevant data sharing protocols) by users within the OBIS-SEAMAP framework (Ocean Biogeographic Information System-Spatial Ecological Analysis of Megavertebrate Populations [30]; http://seamap.env.duke.edu/swot). Thus, the applicability of RMUs to marine turtle conservation and research will be user-dependent, relying on collaboration among users in the community to maintain up-to-date, accurate files.

An important caveat inherent in the RMUs is that we are largely unable to differentiate between true absences (i.e. a species is not identified or is no longer present in an area despite thorough search effort) and absence due to lack of monitoring or reporting. Clearly, distributions of nesting sites and other types of information are biased by areas of high monitoring effort and reporting (e.g. Wider Caribbean region; [31]). In this vein, the maps and RMUs generally could be used in gap analyses to identify areas toward which enhanced census efforts should be directed in order to improve inter-regional comparisons of marine turtle distribution patterns. For example, the distribution of putative RMUs illustrates gaps in scientific understanding of marine turtle biogeography in much of the Indian Ocean, and biogeography of hawksbill turtles in particular (Figs. 1, 2, 3, 4, 5, 6, 7).

In addition to identifying data gaps, a major advantage of the RMU approach is the potential to connect impacts of particular threats to biologically relevant units and their associated demographic characteristics. For example, because marine turtle populations occur in terrestrial and marine habitats, the RMU tool provides the ability to overlay the geographic extent of not only nesting beach threats along a particular coastline (e.g. coastal development) to the particular marine turtle nesting sites and stocks that would be impacted, but also threats across a wider ocean area (e.g. fisheries bycatch) that could impact several nesting stocks and broader population units simultaneously [12]. Furthermore, this system allows identification of spatial overlaps among RMUs – within and among species – which are important areas for conservation because threats impacting multiple RMUs might warrant different management attention than threats acting on a single RMU. Ultimately, although RMUs will not be equally valuable to conservation efforts in all regions, this framework is intended for setting conservation priorities at different levels of spatial and biological organization for marine turtles on a global scale. Below, we outline other potential applications of RMUs to marine turtle conservation and research.

### Potential applications of RMUs

#### Identification essential habitats for marine turtles

Characterization of heavily-utilized areas for marine turtles at different spatial scales is fundamental for creating effective conservation strategies [12], [32]–[34]. One straightforward application of RMUs is to identifying important geographic areas for marine turtle populations in terms of determination of presence, density, and richness. For example, geographic regions that host high densities of nesting sites, possibly of multiple species and/or genetic stocks, could merit investment of conservation resources. Demographic information included in the RMU metadata could also be incorporated in these evaluations to account for abundance and trends among sites and regions.

Likewise, areas of overlap among genetic stocks or geographic distributions, as well as regional variation in population trends at various spatial or biological scales could also be identified using RMUs. Although reproduction areas are relatively discrete geographically and genetically, foraging areas for marine turtles of all life stages host con-specific individuals from multiple stocks and geographic locations [2], [10], [28], [35]–[38]. Therefore, characterizing the connectivity among multiple nesting sites and multiple foraging areas – i.e. ‘many-to-many’ relationships [10] – is necessary for holistically assessing demographic trends and conservation effectiveness [38], [39].

With this in mind, one next step for refining RMUs would be to expand on the genetic stocks based on nesting sites by spatially characterizing at-sea mixed-stock foraging or developmental areas [9], [10], [28], [40], [41]. Moreover, weighting distribution layers according to a relative measure of proportional habitat use (e.g. kernel densities, home ranges) – incorporating both spatial and temporal information – to distinguish among high-use areas and fringe habitats would be another useful extension of the RMU framework. Although incorporating and mapping this information would be challenging given data currently available in published research studies, this step would dramatically improve the ability of RMUs to facilitate identification of marine turtle habitats in relation to other georeferenced information of threats or environmental factors.

#### Improved definition of genetic stock distributions

Despite the emphasis on genetic resolution of MUs, reliance on population structure derived from such analyses can be inadequate for management [4], [42]. Failure to detect population units with genetic markers does not necessarily mean that no management-relevant structuring exists. For example, a newly colonized nesting beach might host turtles that are genetically indistinguishable from the parent (source) population, yet the new nesting cohort is geographically isolated from the parent population. These limitations highlight the need for alternative types of information to make informed MU designations [20].

Along these lines, several studies have proposed or identified geographic or environmental barriers to migration of marine turtles among nesting sites or foraging areas that appear to result in significant population (but not necessarily genetic) differentiation. The distance between nesting sites that results in isolated nesting stocks appears to vary within [2], [43] and among species [13], [44]. Based on identification of 17 distinct green turtle genetic stocks among 27 nesting sites sampled in Australasia, Dethmers et al. [13] proposed that nesting sites separated by more than 500 km are likely to host isolated nesting populations; in contrast, undifferentiated leatherback (*Dermochelys coriacea*) genetic stocks span thousands of kilometers of coastlines within geographic regions [44]. Furthermore, recent studies have described spatial distributions of genetic stocks in foraging and developmental areas at-sea based on local and regional ocean current patterns in the Wider Caribbean [28], [37], Atlantic Ocean [40], Mediterranean Sea [45], and Pacific Ocean [46], [47].

Although barriers to gene flow have not been defined in most cases, these studies illustrate geographic and environmental influences on marine turtle population structures that can be tested within the RMU framework, because nesting sites as well as known genetic stocks are georeferenced. Thus, by applying oceanographic or other physical information as well as relatively simple distance buffering analyses to the RMU files, researchers can test hypotheses about spatial distributions of genetic stocks within and across geographic regions. Resulting distributions of population segments would allow more flexibility in defining MUs than those derived solely from quantitative analyses of genetic differentiation, and would also provide clear objectives for further analyses.

#### Conservation status assessments

Evaluation of species or population status, including population sizes and trends, as well as threats and relevant biological information, is a prerequisite for prioritizing conservation resources. However, because defining the relevant biological or geographic scale at which to conduct assessments and make recommendations is always a fundamental question in these processes, RMUs are a valuable resource to guide how populations of marine turtle species are assessed.

For example, the *IUCN Red List of Threatened Species* represents the only globally accepted system for evaluating extinction risk for species, but the IUCN/SSC Marine Turtle Specialist Group (MTSG) has debated the utility and validity of a global classification system for marine turtles, and has advocated for regional assessments (see [48] for review). A survey of MTSG members from 23 countries revealed that nearly 90% of respondents believed that regional assessments should use either ‘new MTSG criteria’ or ‘flexible, non-standardized criteria’ [48]. However, there was less consensus among members about the appropriate population segment upon which to base regional assessments, with nearly 50% of respondents stating “by geographic region,” ∼30% stating “by genetic stocks,” and ∼20% stating “by nesting sites,” illustrating the challenges inherent in defining management units for marine turtles. Utilizing the RMU framework – which contains information at each of those spatial and biological scales – within or in conjunction with the *Red List* assessment process could address some of the controversy within the MTSG regarding the application of a single global listing for geographically variable marine turtle species by providing regional units for assessment [48], [49].

Another example of incorporating population differences in conservation status assessments is the listing process under the U.S. Endangered Species Act (ESA). The ESA provides for designation and listing of Distinct Population Segments (DPS) of a species based on ‘discreteness’ and ‘significance’ of that segment [50], [51]. In a recent Biological Review of loggerhead turtles (*Caretta caretta*) for purposes of evaluating the species' current Threatened listing status on ESA, nine DPSs were defined, with distinct recommendations for each DPS [52]. Not surprisingly, the process by which DPSs are defined is very similar to that used to define RMUs, as both depend on biogeographical information from genetics, distribution, movements, and demographics. There was nearly complete agreement between the loggerhead DPSs and our RMU designations; the two schemes essentially differed only in how putative RMUs are handled [52] (Table 1). This result provides further support for the validity of the RMU framework for intra-specific conservation assessments.

It is important to note that in geographic regions where there are existing systems for effectively identifying marine turtle population segments to which to target conservation efforts, such as well-defined breeding populations in eastern Australia [13], [16], [18], [21]–[23], RMUs will be of limited utility. In contrast, RMUs will be most appropriately applied to areas hosting several populations or stocks, possibly of multiple species, especially in regions with relatively little available information. Thus, RMUs are designed to be coarse yet flexible enough to be applicable anywhere in the world, rather than being restricted to areas with the best available information, but can be refined in the future when new information becomes available.

#### Marine spatial planning and applications to similar species with complex life histories

To optimize ecosystem function, especially in areas where multiple activities by multiple nations occur, ecosystem-based marine management approaches should be designed to preserve marine biodiversity, keystone species, and biological connectivity among marine habitats [1], [53]. Thus, detailed characterizations of distributions and natural histories of key species (e.g. keystones, top predators, ecosystem engineers) are instrumental to guiding exercises in marine spatial planning [54].

RMUs provide explicit, ecologically based spatial and demographic information about geographic distributions of marine turtle populations that could be integrated with other georeferenced layers, including human activities subject to management (e.g. fisheries operations, hydrocarbon extraction, coastal development, shipping, etc.). Furthermore, the RMU concept could be applied easily to multi-scale biogeographical characterization of other marine megafauna species with similar life history traits and broad, complex distributions. As with marine turtles, identification of high-use habitats, connectivity between breeding and feeding areas, as well as overlapping distributions of distinct populations is extremely important for designing appropriate management schemes for these species [55]–[58].

#### Conclusions

The novel RMU framework synthesizes available biogeographical information on globally distributed and imperiled marine turtles into a multi-scaled, geospatial tool for which we envision numerous pertinent applications for marine turtle conservation and research. For example, the MTSG Burning Issues Working Group is utilizing RMUs as the basis for status assessments in a developing process of global conservation priority setting for marine turtles. The RMU classification system is consistent with endangered species laws in the United States and elsewhere, and could provide the IUCN-MTSG with a way forward for regional evaluations of extinction risk.

Although species are predominantly used as the currency for evaluating and prioritizing conservation efforts (e.g. *IUCN Red List*, Alliance for Zero Extinction, Conservation ‘Hotspots’), in the case of globally distributed species like marine turtles, regional population segments occupy distinct ecological roles and thus merit conservation attention, because extinction of an RMU would represent the loss of the species' ecological role within an entire region and ecosystems therein [59]. By defining populations according to ecological characteristics, the RMU approach implies the inherent importance of each RMU as an independent conservation unit, which species-focused conservation approaches might fail to recognize.

We emphasize that the resolution of RMUs does not detract from the treatment of nesting populations as management units. Abundant historical, mark-recapture, and genetic data indicate that nesting populations will rise and fall as independent demographic units. The value of RMUs is in the recognition of regional groupings of marine turtles that overlap in feeding and nursery habitats, exchange genetic material, and face common threats at sea. Therefore, the RMU framework is a solution to the challenge of how to organize marine turtles into units of protection above the level of nesting populations, but below the level of species, within regional entities that might be on independent evolutionary trajectories. Finally, the RMU system is neither static nor proprietary, but rather is iterative and user-driven. We encourage broad and creative engagement with and application of this tool, whose long-term accuracy and efficacy will rely on updates, edits, and improvements arising from user interactions.

## Supporting Information

Table S1 Summary of Regional Management Units (RMUs) for marine turtles worldwide, including number of nesting sites and genetic stocks contained within each RMU.
(DOC)

Appendix S1 Complete list of SWOT – The State of the World's Sea Turtles data providers.
(XLS)

Appendix S2 Metadata associated with each layer synthesized to generate Regional Management Units.
(XLS)
