# Supplementary material for: Regional Management Units for Marine Turtles: A Novel Framework for Prioritizing Conservation and Research across Multiple Scales
Source: PLoS One. 2010 Dec 17;5(12):e15465. doi: 10.1371/journal.pone.0015465 (PMC3003737; doi:10.1371/journal.pone.0015465)
Supplement: Table S1 — Summary of Regional Management Units (RMUs) for marine turtles worldwide, including number of nesting sites and genetic stocks contained within each RMU. (DOC) [file pone.0015465.s001.doc]

**Regional Management Units for marine turtles: A novel framework for prioritizing conservation and research across multiple scales**

Bryan P. Wallace^1,2,3^* et al.

^1^IUCN/SSC Marine Turtle Specialist Group – Burning Issues Working Group, ^2^Global Marine Division, Conservation International, Arlington, VA, USA, ^3^Center for Marine Conservation, Duke University, Beaufort, NC, USA

***Corresponding author: b.wallace@conservation.org**

**Supplementary Information**

**Table S1.** Summary of Regional Management Units (RMUs) for marine turtles worldwide. Number of nesting sites quantified since 2000, genetic stocks (both mitochondrial [mtDNA] and nuclear [nDNA]) within the geographic extent of each RMUs presented (Note: nesting sites and genetic stocks are counted within each RMU in which they appear, so some are counted more than once). For *L. olivacea*, number of nesting sites and genetic stocks are the same for arribada and non-arribada RMUs because geographic boundaries of RMUs are identical (see text for more information). RMUs in italics denote “putative RMUs,” or those that contained nesting sites but had no accompanying biogeographical information with which to draw boundaries. ND: no data. (* denotes that although nesting sites have not been sampled for genetics, all *L. kempii* individuals are presumed to belong to same stock and RMU.)

| **species** | **ocean** | **region** | **no. nesting sites** | **no. mtDNA stocks** | **no. nDNA stocks** |
| --- | --- | --- | --- | --- | --- |
| *Caretta caretta* | Atlantic | Northeast | 10 | 1 | ND |
| *Caretta caretta* | Atlantic | Northwest | 332 | 5 | 1 |
| *Caretta caretta* | Atlantic | Southwest | 95 | 1 | 1 |
| *Caretta caretta* | *Indian* | *Northeast* | 12 | ND | ND |
| *Caretta caretta* | Indian | Northwest | 3 | 1 | ND |
| *Caretta caretta* | Indian | Southeast | 4 | 1 | 1 |
| *Caretta caretta* | Indian | Southwest | 12 | 1 | ND |
| *Caretta caretta* | Mediterranean |  | 82 | 4 | 4 |
| *Caretta caretta* | Pacific | North | 46 | 1 | ND |
| *Caretta caretta* | Pacific | South | 30 | 1 | 1 |
| *Chelonia mydas* | Atlantic | Central | 1 | 1 | 1 |
| *Chelonia mydas* | Atlantic | Northwest | 213 | 2 | 1 |
| *Chelonia mydas* | Atlantic | South Caribbean | 197 | 1 | 1 |
| *Chelonia mydas* | Atlantic | East | 16 | 2 | 1 |
| *Chelonia mydas* | Atlantic | Southwest | 20 | 2 | 1 |
| *Chelonia mydas* | Indian | Northeast | 18 | 1 | ND |
| *Chelonia mydas* | Indian | Northwest | 94 | 1 | 1 |
| *Chelonia mydas* | Indian | Southeast | 9 | 2 | 2 |
| *Chelonia mydas* | Indian | Southwest | 68 | 3 | ND |
| *Chelonia mydas* | Mediterranean |  | 14 | 1 | 1 |
| *Chelonia mydas* | Pacific | East | 30 | 2 | 1 |
| *Chelonia mydas* | Pacific | North Central | 1 | 1 | 1 |
| *Chelonia mydas* | Pacific | Northwest | 8 | 1 | 1 |
| *Chelonia mydas* | Pacific | South Central | 164 | ND | ND |
| *Chelonia mydas* | Pacific | Southwest | 34 | 7 | 3 |
| *Chelonia mydas* | Pacific | West | 24 | 5 | ND |
| *Chelonia mydas* | Pacific | West Central | 12 | 1 | ND |
| *Dermochelys coriacea* | Atlantic | Northwest | 422 | 4 | 3 |
| *Dermochelys coriacea* | Atlantic | Southwest | 11 | 1 | ND |
| *Dermochelys coriacea* | Atlantic | Southeast | 45 | ND | ND |
| *Dermochelys coriacea* | Indian | Northeast | 63 | ND | ND |
| *Dermochelys coriacea* | Indian | Southwest | 8 | 1 | 1 |
| *Dermochelys coriacea* | Pacific | East | 40 | 1 | 1 |
| *Dermochelys coriacea* | Pacific | West | 63 | 2 | 1 |
| *Eretmochelys imbricata* | Atlantic | East | 19 | 1 | ND |
| *Eretmochelys imbricata* | Atlantic | Southwest | 49 | 1 | ND |
| *Eretmochelys imbricata* | Atlantic | West | 819 | 9 | ND |
| *Eretmochelys imbricata* | *Indian* | *Northeast* | 74 | ND | ND |
| *Eretmochelys imbricata* | *Indian* | *Northwest* | 38 | 2 | ND |
| *Eretmochelys imbricata* | *Indian* | *Southeast* | 6 | 1 | 1 |
| *Eretmochelys imbricata* | *Indian* | *Southwest* | 59 | 1 | ND |
| *Eretmochelys imbricata* | *Pacific* | *North Central* | 8 | ND | ND |
| *Eretmochelys imbricata* | *Pacific* | *South Central* | 136 | ND | ND |
| *Eretmochelys imbricata* | *Pacific* | *West* | 31 | 2 | ND |
| *Eretmochelys imbricata* | *Pacific* | *West Central* | 29 | ND | ND |
| *Eretmochelys imbricata* | Pacific | East | 41 | ND | ND |
| *Eretmochelys imbricata* | Pacific | Southwest | 24 | 2 | 1 |
| *Lepidochelys olivacea* | Atlantic | East | 34 | 1 | 1 |
| *Lepidochelys olivacea* | Atlantic | West | 57 | 1 | 1 |
| *Lepidochelys olivacea* | Indian | Northeast | 46 | 2 | ND |
| *Lepidochelys olivacea* | Indian | Northeast (arribadas) | 46 | 2 | ND |
| *Lepidochelys olivacea* | *Indian* | *West* | 82 | 2 | ND |
| *Lepidochelys olivacea* | Pacific | West | 65 | 1 | ND |
| *Lepidochelys olivacea* | Pacific | East | 135 | 2 | 1 |
| *Lepidochelys olivacea* | Pacific | East (arribadas) | 135 | 2 | 1 |
| *Lepidochelys kempii* | Atlantic | Northwest | 30 | 1* | 1* |
| *Natator depressus* | Indian | Southeast | 252 | 3 | ND |
| *Natator depressus* | Pacific | Southwest | 35 | 2 | ND |
